# Prediction of the shear capacity of ultrahigh-performance concrete beams using neural network and genetic algorithm

**DOI:** 10.1038/s41598-023-29342-0

**Published:** 2023-02-07

**Authors:** Rui Hou, Qi Hou

**Affiliations:** 1 School of Civil and Architecture Engineering, Hunan Institute of Technology, Hengyang, 421002 China; 2 Hunan Hongli Civil Engineering Inspection and Testing Co., Ltd., ChangSha, Hunan Province China

**Keywords:** Civil engineering, Engineering

## Abstract

Currently, concrete structures have increasingly higher requirements for the shear capacity of beams, and ultrahigh-performance concrete (UHPC) beams are increasingly widely used. To facilitate the design of UHPC beams, this paper constructs a UHPC beam shear strength prediction model. First, static shear tests were conducted on 6 UHPC beam specimens with a length of 2 m and a cross-sectional size of 200 mm × 300 mm to explore the effects of the UHPC strength, shear span ratio, hoop ratio, and steel fiber content on the shear resistance and failure morphology of the UHPC beams. Based on the results of this study and a static load experiment of 102 UHPC beams in the literature, the construction includes the shear span ratio (λ), beam section width (b), beam section height (h), hoop ratio (*ρ*_SV_), UHPC compressive strength (*f*_c_), steel fiber volume fraction (*V*_f_), and the UHPC beam shear capacity (*V*_ex_) 7 parameter database. Based on the construction of the database, 1200 BPNN models were trained through trial and error. The models were evaluated using the correlation coefficient R, root mean square error RMSE, and a20-index indicators, and the optimal BPNN model (6-15-8-1) was determined based on the ranking of RMSE. After the optimal BPNN is optimized by a genetic algorithm, the prediction performance of the model is improved. The correlation coefficient between the predicted value and the experimental value is R^2^ = 0.98667, and RMSE = 7.38. This model can reliably predict the shear strength of UHPC beams and provide designers with a reference for the design of UHPC beams. Finally, after sensitivity analysis, the influence of each input parameter on the UHPC shear capacity is determined.

## Introduction

Concrete is one of the most basic building materials with good compressive performance, but its shear strength and tensile strength are often low^1,2^. With the development of super high-rise structures with large spans, many scholars have studied UHPC^3,4^. UHPC is a new type of building material that shows excellent performance in strength, toughness, and durability, making it widely used in long-span, thin-walled, and curved structures^5–7^.

UHPC removes the coarse aggregates in traditional concrete, uses quartz sand as fine aggregates, increases the content of active powders (silica fume, fly ash, slag powder) and is configured with superplasticizers, which can be self-flowing and dense. Forming and no segregation bleeding phenomena occur while ensuring higher compressive strength and tensile strength^8^, with excellent durability and impermeability^9^. In addition, the UHPC matrix contains evenly distributed steel fibers, which confer high toughness to UHPC^10,11^. Therefore, UHPC is considered the most promising material for extreme environments. In recent years, many scholars have applied UHPC to structural reinforcement. Based on the strong adhesion between the material interfaces, it acts on the surface of the object to be reinforced, changing the cross-section of the structure and participating in the force to enhance the performance of the reinforced object. Karthik et al.^12^ and Prem^13^ studied the flexural performance of damaged reinforced concrete (RC) beams strengthened by UHPC overlays, and Bahraq et al.^14^ studied ultrahigh performance fibers. The shear performance of UHPC-reinforced RC beams shows that the flexural and shear bearing capacity of RC beams are significantly improved, the failure mode changes from brittleness to toughness, and the reinforced RC beams have higher stiffness. Chen Cheng et al.^15^ used UHPC- and fiber-reinforced polymer (FRP) composite materials to strengthen corroded reinforced concrete beams for shear resistance. Experimental studies have shown that, in addition to the significant increase in the shear bearing capacity of reinforced concrete beams, the introduction of a thin CFRP mesh into ultrahigh performance concrete suppresses the formation of shear cracks and reduces the width of concrete cracks.

Because UHPC has high strength, high toughness and high durability that ordinary concrete can hardly reach^16,17^, theoretically, it can effectively reduce the size of beam members without reducing the shear capacity of the beam structure^18^. Many scholars have conducted experimental studies on the structural response of UHPC beams^19–22^. The results show that, compared with ordinary concrete, UHPC with steel fibers can significantly improve the cracking stiffness and bending resistance of the beam. To study the shear resistance of UHPC beams, many studies have proposed empirical formulas to estimate the shear strength of UHPC beams^23–26^, but the experimental results and the results of the prediction formula often differ^27^. These empirical formulas are aimed at specific experimental research. In addition, the application of the finite element simulation method has strong limitations, and the cost of laboratory experiments is high, so a reliable mathematical model is needed to predict the shear strength of UHPC beams.

In recent years, artificial intelligence machine learning technology has been widely used in structural engineering, such as material performance prediction^28–31^, structural health detection^32^, and other fields. Machine learning technology can solve problems that cannot be solved by traditional methods. Among them, artificial neural networks (ANNs), support vector machines (SVMs), and random forests (RFs) are widely used^33–35^. Mangalathu et al.^36^ used two machine learning models, neural networks and random forests, for bridge damage detection and the seismic risk of tilted bridges. Dao^37^ and others used an adaptive network-based fuzzy inference system (ANFIS), artificial neural network (ANN), and support vector machine (SVM) to predict the compressive strength of polymer concrete. Adhikary et al.^38^ used an ANN to predict the shear strength of SFRC beams and showed a good predictive effect. Lee^39^ et al. used an ANN to predict the shear strength of fiber-reinforced concrete beams. The results show that the ANN is better than the empirical formula. Vu et al.^40^ developed a combined least squares support vector machine model (LS-SVM) to predict the punching shear capacity of concrete slabs. Chopra^41^ and others used decision trees, random forests, neural networks, and other models to predict the compressive strength of concrete. The results show that the prediction effect of the neural network is the best. Momeniet al.^42^ established a GA-based ANN to predict the pile bearing capacity and suggested that the implementation of GA-based ANN models as a highly reliable, efficient, and practical tool in predicting the pile bearing capacity is advantageous.

Mansour et al.^43^ built an ANN model using the available test data of 176 RC beams collected from the technical literature. The results show that ANNs have strong potential as a feasible tool for predicting the ultimate shear strength of RC beams with transverse reinforcement within the range of input parameters considered. Hosseini^44^ recognized the ability of an artificial neural network, which was trained based on a genetic algorithm, to predict the shear capacity of reinforced concrete beams strengthened with side-bonded fiber-reinforced polymer (FRP). The reason for the difficulty is the complex mechanics of the problem, where the steel fibers affect the different shear-carrying mechanisms. Since this problem is still not fully understood, Abambres et al.^45^ proposed the use of artificial intelligence (AI) to derive an expression based on the available experimental data. Zhang et al.^46^ proposed the SVR-GA model as an applicable and robust computer aid technology for modelling RC deep beam shear strength that contributes to the base knowledge of material and structural engineering perspectives. Feng et al.^47^ established an analysis model of the shear capacity of prestressed ultrahigh performance concrete (UHPC) beams under the combined action of bending and shearing based on the modified compression field theory and by considering the unique material constitutive relation of UHPC.

To facilitate the structural design of UHPC beams, this paper applies one of the most effective machine learning model artificial neural networks to predict the shear capacity of UHPC beams and uses genetic algorithms to optimize the neural network model. First, six test beams are made through experimental design, and a static loading test is performed to study the influence of the UHPC strength, shear span ratio and hoop ratio on the shear capacity of UHPC beams. Based on the experimental results and literature research, a model training database containing 102 sets of data is constructed. The database is divided into two parts: 72 (70.59%) data points are used for model training, and 30 (29.41%) data points are used for model testing. Finally, the test results and prediction results are statistically analysed to evaluate the prediction performance of the ANN model for the UHPC beam shear capacity.

## Experiment plan

### Specimen design

**Figure 1:**
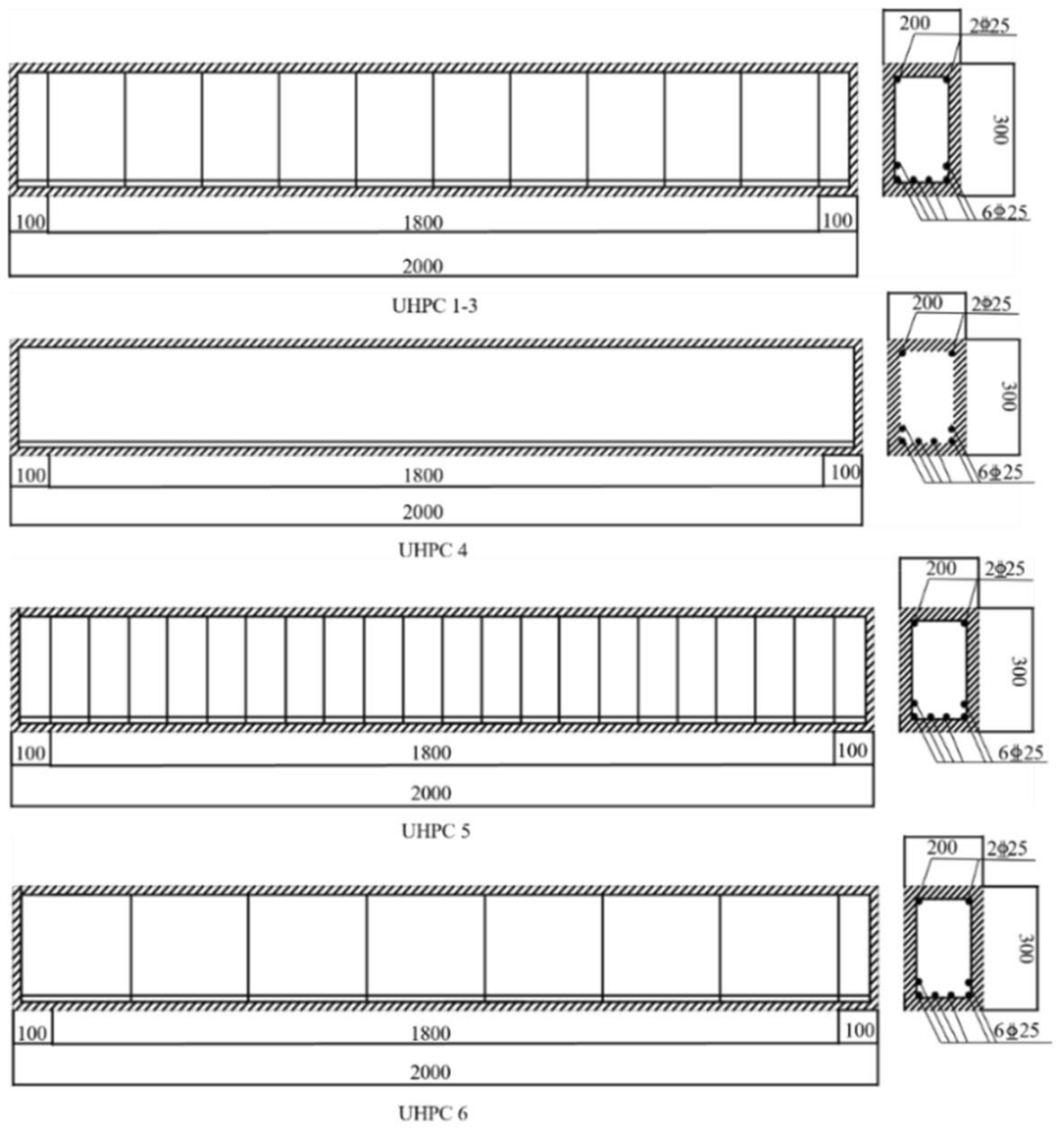
UHPC beam reinforcement diagram.

A total of 6 test beams were designed, and the shear span ratio (λ), UHPC strength (*f*_c_), reinforcement ratio (*ρ*_SV_), and steel fiber volume ratio (*V*_f_) were selected as the control parameters for the static loading test. The specimen number is UHPC-1 ~ 6, the cross-sectional dimension of the specimen is 200 mm × 300 mm, and the length is 2000 mm. The shear span ratio was set to 1.0, 1.5, and 2.0, and *V*_f_ was set to 0, 1, 2, and 3%. The longitudinal bars and stirrups of the specimens are made of HRB400 threaded bars, the diameters of the tension bars and compression bars are both 25 mm, and the diameter of the stirrups is 8 mm. The specific parameters of the specimen are shown in Table 1, and the reinforcement of the specimen is shown in Fig. 1.

**Table 1:** Specimen design parameters.

Test specimen number	Sectional dimension/mm	λ	f_c_	ρ_sv_/%	V_f__(%)_
UHPC-1	200 × 300	1.0	110	3.2	0
UHPC-2	200 × 300	1.5	110	3.2	1
UHPC-3	200 × 300	1.5	125	3.2	2
UHPC-4	200 × 300	2.0	125	0	3
UHPC-5	200 × 300	2.0	150	4.5	0
UHPC-6	200 × 300	2.0	110	2.5	1

The pouring test beam was made of UHPC dry mix with a fixed mix ratio, and UHPC of different strength grades was prepared by controlling the curing conditions. All test beams were horizontally poured. The prefabricated steel framework was put into the wooden formwork and then moulded and poured once, and the corresponding batch of UHPC test blocks was reserved. After the test beam was left in the room for 24 h, the mould was removed. After the mold was removed, the test beam and the test blocks were cured under the same conditions. The UHPC120 test beam and test block were cured at room temperature, and the UHPC150 test beam and test block were first cured with 80 °C hot water for 24 h and then cured at room temperature.

### Material mechanical properties

All stirrups and longitudinal reinforcements of the specimens were HRB400 threaded steel bars, the diameter of the longitudinal reinforcement was 25 mm, and the diameter of the stirrup was 8 mm. The mechanical properties of the steel bars are shown in Table 2.

**Table 2:** Mechanical property of steel bars.

Steel bar type	Yield strength/MPa	Tensile strength/MPa	Elastic modulus/GPa
HRB400	441.0	635.85	2.0 × 10^5^

The mechanical properties of each batch of UHPC test blocks cured under the same conditions were tested, and the cubic compressive strength *f*_cu_, compressive strength *f*_c_, splitting strength *f*_ts_, and elastic modulus *E*_c_ were measured. The sizes of the test blocks used were 100 mm × 100 mm × 100 mm, 150 mm × 150 mm × 150 mm, 150 mm × 150 mm × 300 mm, and 100 mm × 100 mm × 300 mm. The test results are shown in Table 3.

**Table 3:** Mechanical properties of UHPC.

UHPC strength	*f*_cu_/MPa	*f*_c_/MPa	*f*_ts_/MPa	*E*_c_/GPa
R120	121.8	104.1	8.3	42.3
R150	152.4	128.6	9.5	47.5

### Loading method and measurement

**Figure 2:**
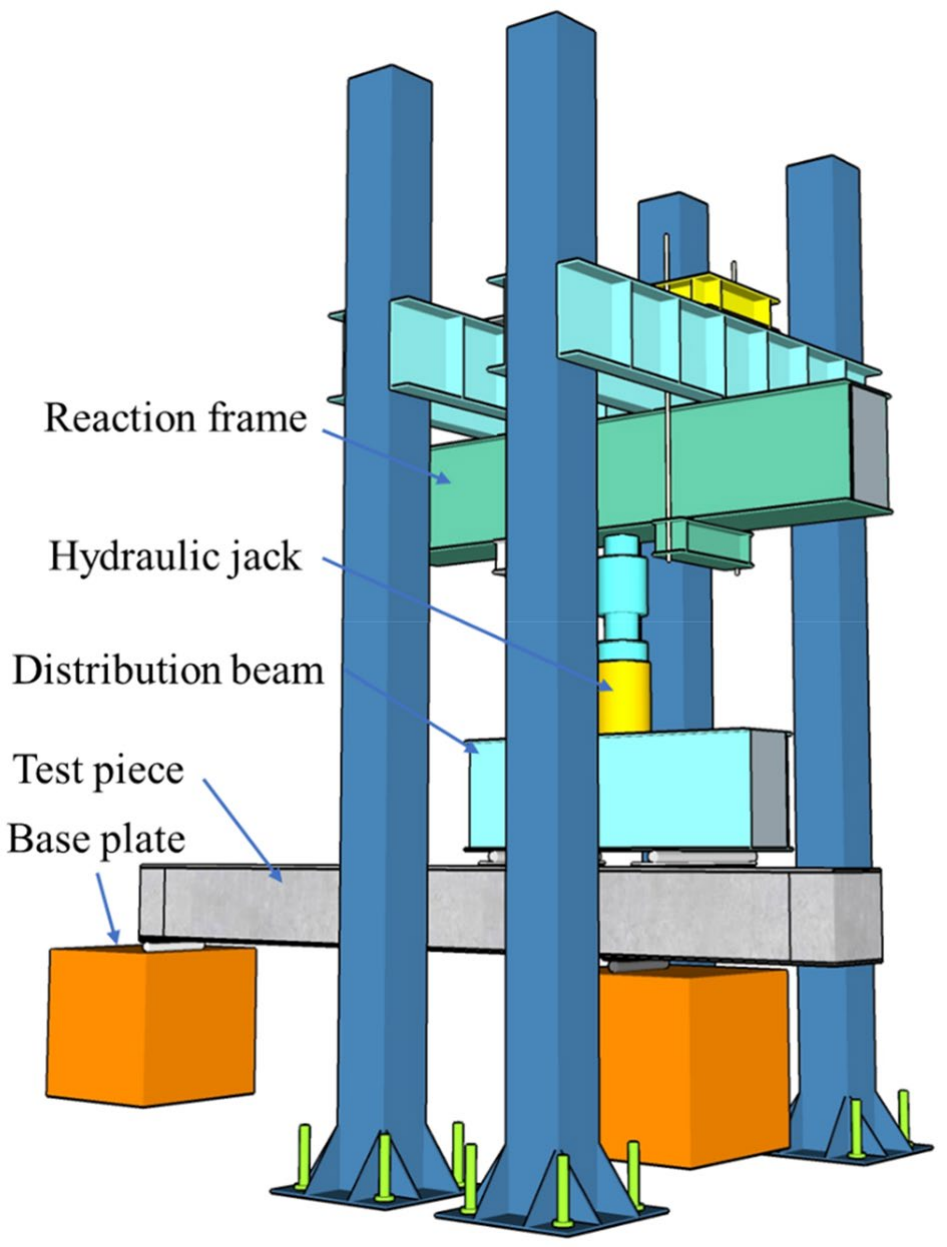
Schematic diagram of test loading.

**Figure 3:**
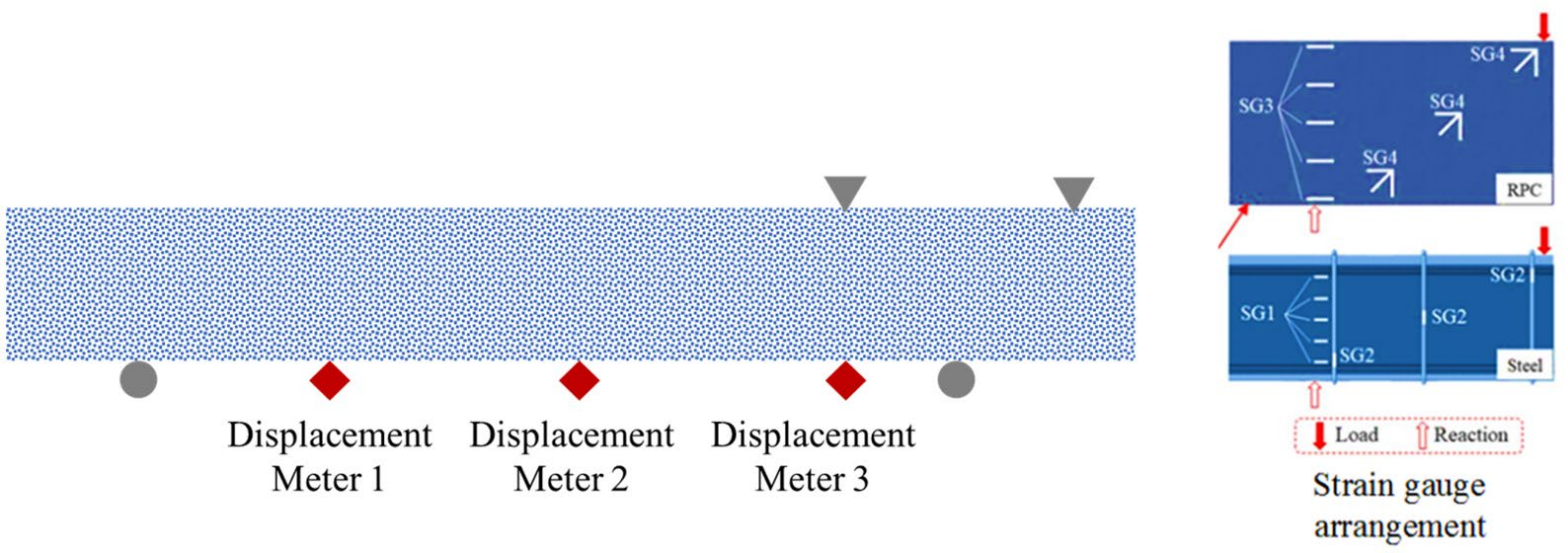
Arrangement of the strain gauge and displacement meter.

An electrohydraulic servo press control system was used in the test, and monotonic continuous load control was used to control the loading. The specific loading situation is shown in Fig. 2. The specimens were loaded in stages. Before the crack width was less than 0.3 mm, the load of each stage was 10% of the calculated value of the ultimate load. When the crack width was greater than 0.3 mm, the load of each level was set to 5% of the calculated value of the ultimate load, and the specimen was loaded until the specimen failed. Strain data were collected through a static strain test system, and displacement meters were arranged at 1/4, mid-span and 3/4 of the beam. The layouts of the strain gauges and displacement gauges are shown in Fig. 3.

## Test results and discussion

### Shear failure characteristics

**Figure 4:**
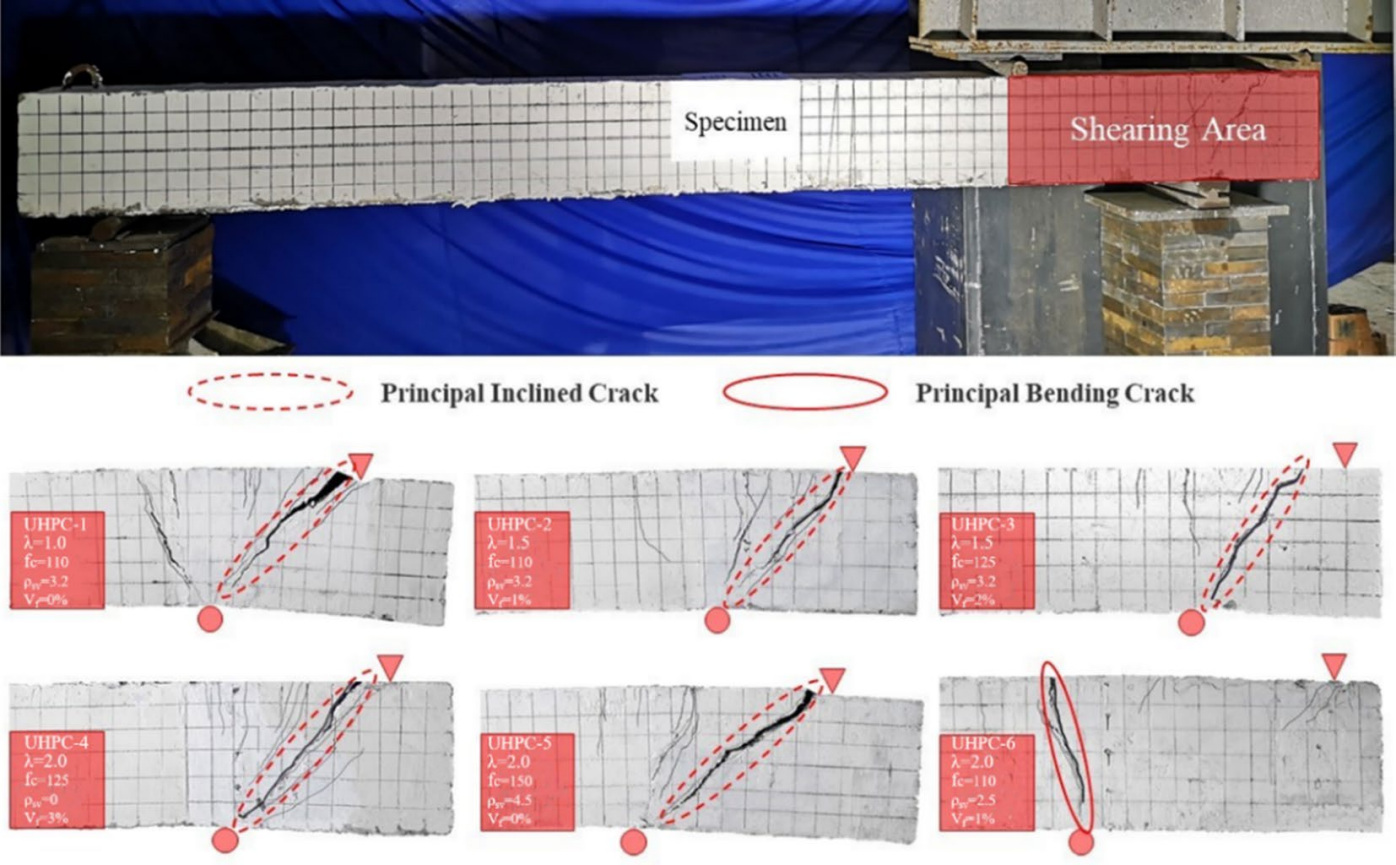
Failure mode of a specimen.

The results show that the six test beams have different degrees of bending and shear failure, and the UHPC-6 test beam shows an obvious bending failure mode. The failure mode of the test beam is shown in Fig. 4.

According to the failure process of the six test beams, the shear failure process of the UHPC beams can be divided into the elastic stage, cracking stage, crack development stage, and failure stage.

#### Elastic stage

From the initial loading stage to the cracking stage, the steel bar and UHPC worked together completely, the strain of the two materials increased linearly, and the increase was small. Taking the UHPC-1 test beam as an example, the strain value of the measuring point of the upper flange of the UHPC beam was 410 με before loading to cracking. The strain values of the upper flange of the other test beams were only several hundred microstrains before cracking. Similarly, the deflection increases linearly in the initial stage of loading.

#### Cracking stage

According to five test beams with shear failure, when loaded to 25–35% of the ultimate load, fine transverse cracks appeared on the upper surface of the UHPC at the bearing, and the strain value would first overflow. As the load continued, slight vertical cracks appeared on the UHPC surface of the beam side. Both sides of the cracks were bending-shear cracks, and the abdominal shear cracks in the shear span section appeared later than the bending-shear cracks, which were distributed on the UHPC surface of the beam side at the axis of gravity of the test beam in the shear span section. When the test beam was in the shear state, the horizontal tensile stress of the element at the top of the beam reached the tensile strength of the UHPC before the main tensile stress of the element near the central axis, and cracks appeared. At this time, the steel bar was still in the elastic deformation stage.

#### Crack development stage

When the load continued to increase, the number of bending and shear cracks continued to increase, but no obvious trend of widening and extending downwards was evident. After abdominal shear cracks appeared, the abdominal shear cracks extended to the loading point and the support as the load increased, and new oblique cracks continued to appear on both sides as the load increased and extended to both ends. When the load reached more than 60% of the ultimate load, new oblique cracks no longer appeared on the surface of the UHPC. The existing oblique cracks began to extend to both ends, but the width did not increase significantly. From the perspective of the deflection development of the cantilever end, UHPC on both sides of the oblique cracks did not rapidly withdraw from work because of the development of oblique cracks. From the distribution and development of cracks, no relative slip occurred between steel and concrete, indicating that the setting of stirrups could realize the effective anchorage between steel and UHPC, and the two could form a good cooperative working mechanism. When the load was loaded to more than 70% of the ultimate load, a critical oblique crack developed among many oblique cracks, the steel fibre exfoliation fracture began to sound inside the test beam, and the sound became increasingly intensive with the continuous increase in load.

#### Failure stages

When the load reached more than 85% of the ultimate load, the UHPC surface could bulge at the support, and signs of UHPC collapse began to appear. At the same time, the critical oblique crack developed into a main oblique crack, and the width of the crack increased significantly with increasing load. When the load increased to 90% of the ultimate load, the steel fibre peeling fracture sound was intensively heard inside the test beam, and the main oblique crack of UHPC on both sides gradually withdrew from work as the crack width increased. The deflection of the cantilever end increased by 2-threefold, indicating that the steel skeleton of the shear span section yielded in a large area. When loading reached the ultimate load, the test beam presented an obvious shear failure mode, and the deflection of the cantilever end increased continuously, so the next loading could not be performed.

### Test results

**Figure 5:**
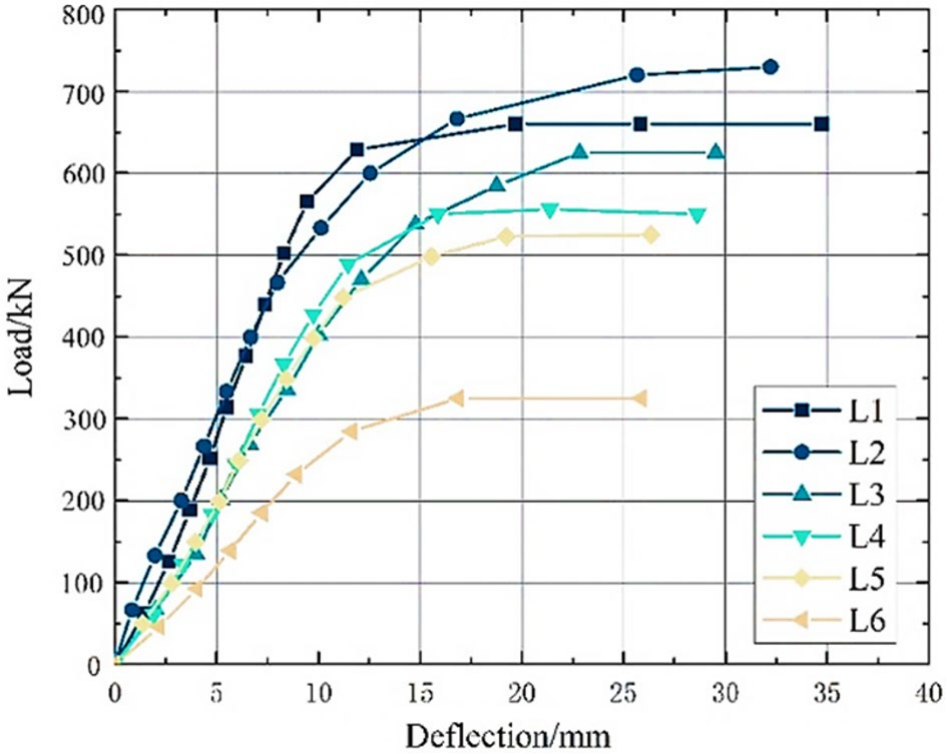
Load‒deflection curve of the test beams’ cantilever.

**Figure 6:**
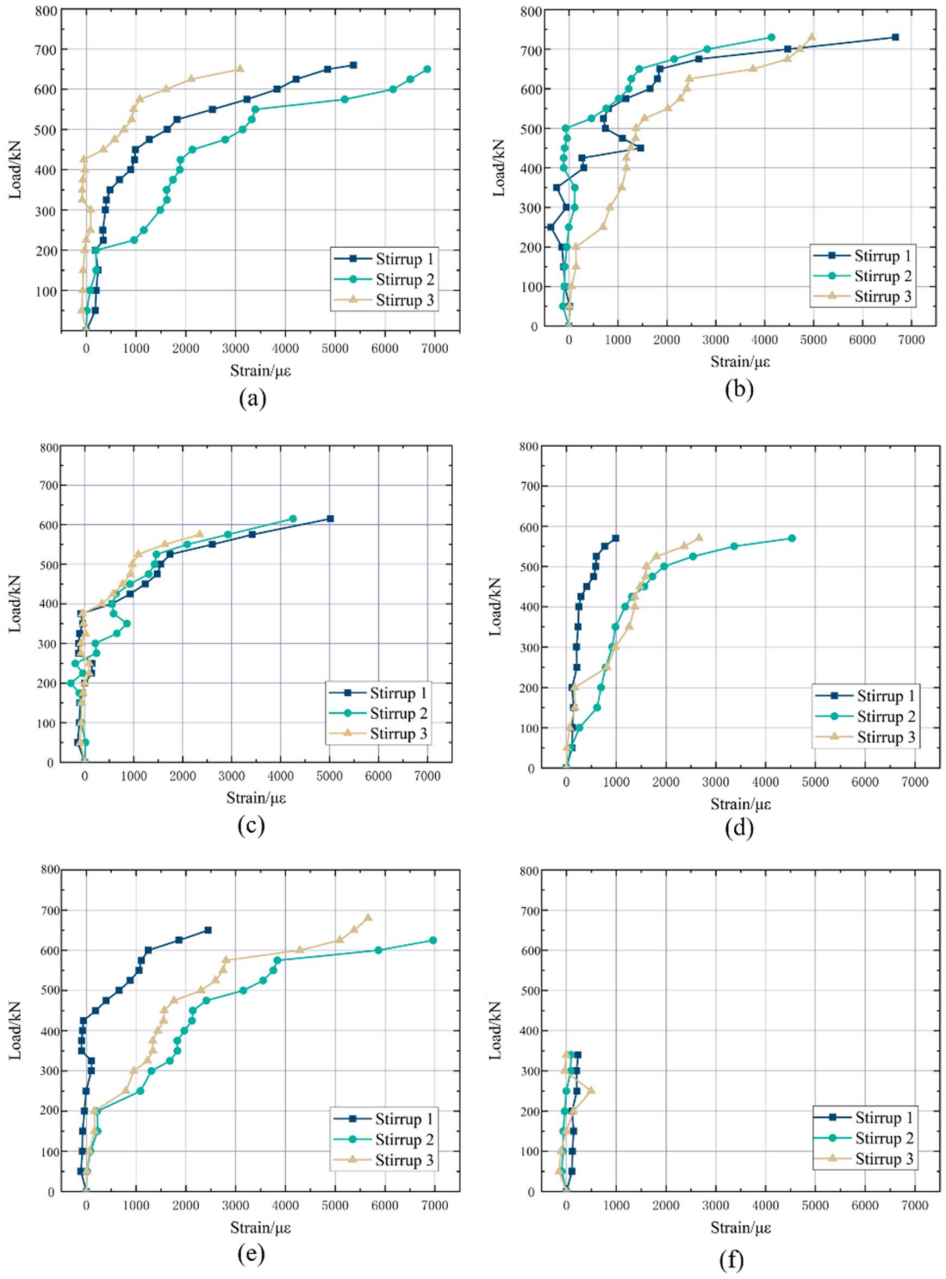
Load-strain curve of stirrups in the test beams’ spear-span area {(**a**) UHPC-1; (**b**) UHPC-2; (**c**) UHPC-3; (**d**) UHPC-4; (**e**) UHPC-5; (**f**) UHPC-6}.

The cracking load P_cr_, ultimate load P_u_ and deflection v_u_ of the six test beams are shown in Table 4. The load and cantilever end deflection curves are shown in Fig. 5, and the load and shear span stirrup strain curves are shown in Fig. 6.

**Table 4:** Cracking load *P*_cr_, ultimate load *P*_u_ and deflection *v*_u_ of the specimens.

Specimen	*P*_cr_/kN	*P*_u_/kN	*v*_u_/mm
UHPC-1	430	660	34.75
UHPC-2	450	710	32.22
UHPC-3	400	625	29.53
UHPC-4	360	570	28.62
UHPC-5	350	525	26.36
UHPC-6	300	650	25.87

## Database establishment

**Figure 7:**
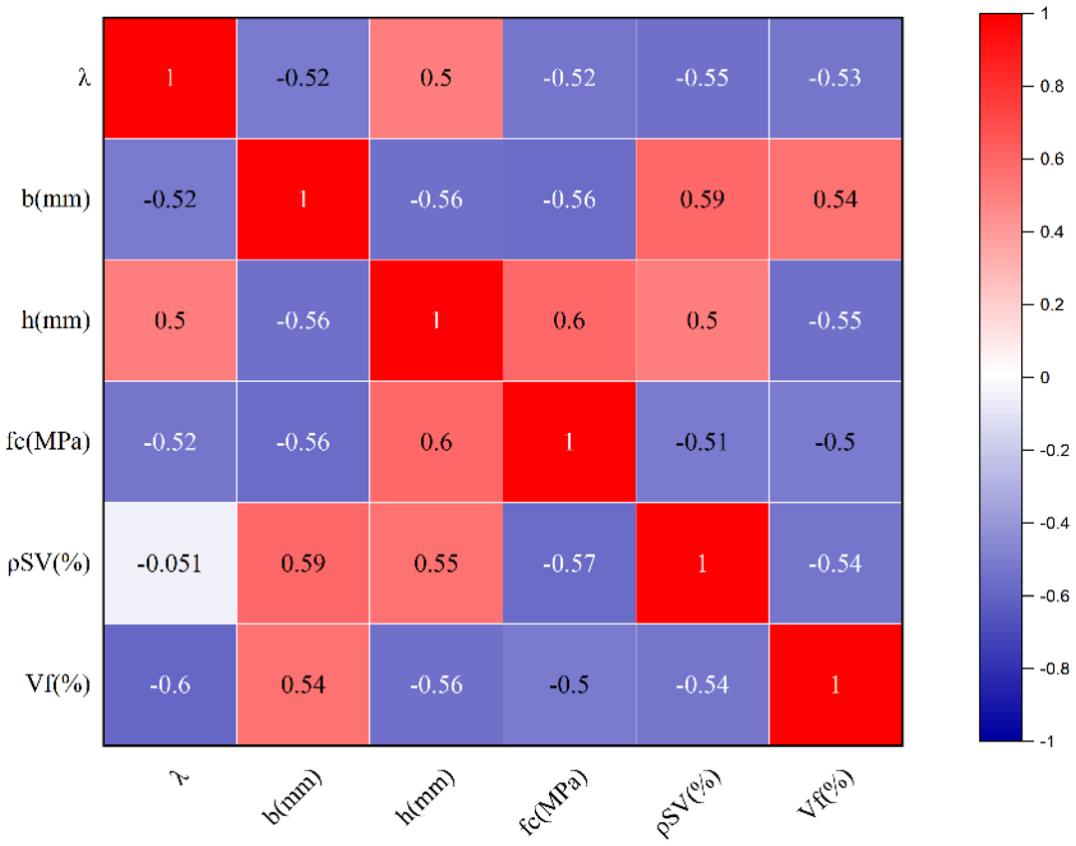
Correlation coefficient matrix of input parameters.

It is evident from previous studies that the choice of input parameters is crucial for training the prediction model. Several factors influence the load-carrying capacity of ultrahigh-performance concrete beams. We determined six main influencing parameters regarding the relevant code standards and previous studies^57–65^: the shear span ratio λ, beam section width b, beam section height h, stirrup ratio ρSV, UHPC compressive strength fc, and steel fiber volume fraction Vf. The correlation matrix between the shear-bearing capacity of UHPC beams and the input parameters is shown in Fig. 7 below. The correlation coefficients of all input parameters are more significant than 0.5, and the correlation is relatively.

Based on the six test beams in this study, the test results of 102 UHPC beams in References^57–65^ were collected. The collected parameters of the specimen included the shear span ratio λ, beam section width b, beam section height h, stirrup ratio *ρ*_SV_, UHPC compressive strength f_c_, steel fiber volume fraction *V*_f_ and shear bearing capacity *V*_ex_, as shown in the Appendix. Voo et al.^48^ reported the results of eight steel fiber-reinforced prestressed concrete beams tested in shear with the test variables being the shear span-to-effective depth ratio and the quantity and type of steel fibers. The shear capacity of the UHPC beam is calculated by using concrete plasticity theory, which is in good agreement with the experimental and literature results. Haibin et al.^49^ used the limit equilibrium method, modified pressure field theory and plasticity theory to analyse the shear capacity of ultrahigh performance concrete beams. This shows that the calculation result of the shear strength by the limit equilibrium method is close to the test value. Lingzhi et al.^50^ carried out a shear performance test on 12 RPC test beams with high strength reinforcement, studied the influence of the shear span ratio, hoop ratio, longitudinal reinforcement ratio, steel fiber volume ratio and other parameters, and proposed a formula for calculating the shear capacity of RPC beams considering the influence coefficient of the section size. Qiang et al.^51^ proposed a formula for calculating the shear capacity considering the plastic coefficient based on the shear test results of 14 reinforced RPC beams, which considered the influence of the shear span ratio, steel fiber content, stirrup ratio, reinforcement ratio and longitudinal reinforcement strength, but it was not suitable for baroclinic failure with a relatively small shear span. Zongcai et al.^52^ proposed an improved pressure field model based on the shear test results of 12 RPC beams with high strength reinforcement and found that the contribution of the Vilar pull resistance of steel fibers to the shear strength reached 40–60%. Aziz et al.^53^ studied the shear strength and behavior of deep beams under two-point loading. The experimental results showed that the compressive strength of concrete and the shear span to depth ratio (a/d) also have a significant effect on the failure load. Lim et al.^54^ conducted shear tests on a simply supported UHPFRC beam with a fiber volume fraction of 1.5% and investigated the shear behavior of the beam with different stirrup spacing. The results showed that a spacing limit of 0.75 d can be allowed for UHPFRC beams. Pu et al.^55^ carried out shear tests on 12 RPC beams with different stirrup ratios, longitudinal reinforcement ratios and shear-span ratios, simulated the stress process of RPC beams after cracking, and analysed the factors affecting the shear capacity of RPC beams based on the softening truss mode. Pourbaba et al.^56^ produced 19 ultrahigh performance RC rectangular beam specimens for shear performance studies and compared the test results with those predicted by ACI 318, RILEM TC 162-TDF, Australian Guidelines and the National Building Code of Iran. Compared with the test values, the predicted shear force obtained by various codes has a safety factor of 10 times.

**Figure 8:**
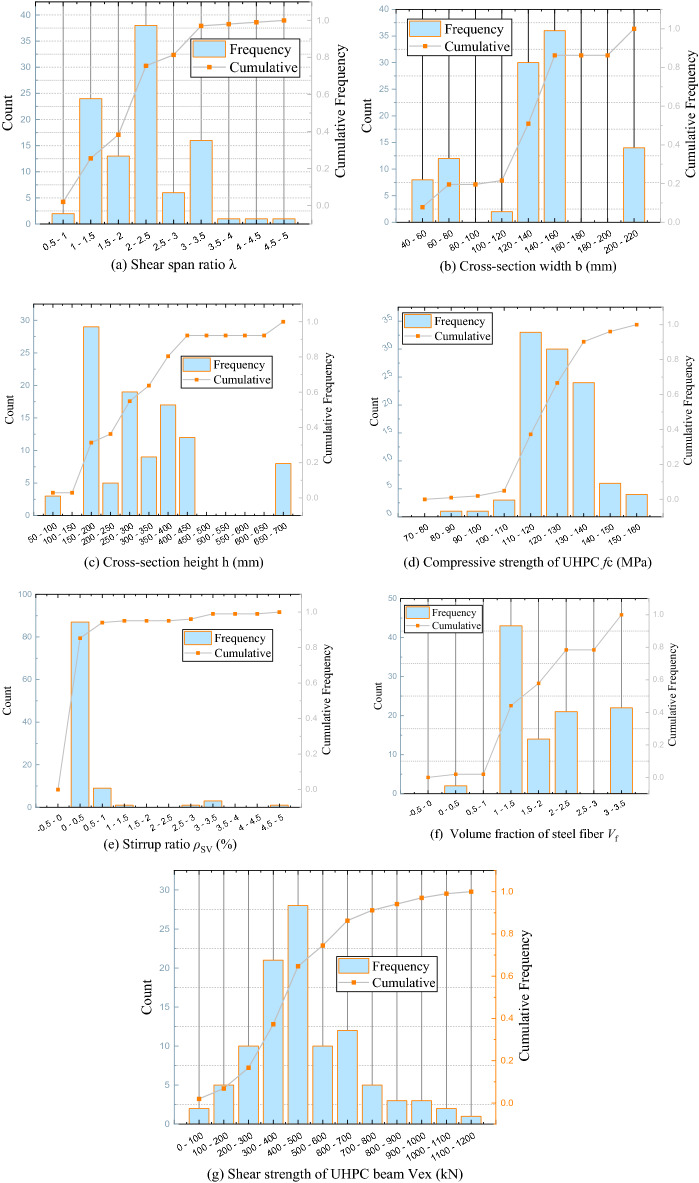
Variable frequency distribution histogram.

Table 5 shows the results of a statistical analysis of the database, including the maximum, minimum, average, and standard deviation. Each parameter of the database had sufficient coverage and covered most possible situations, which is beneficial to the training of the ANN model. Figure 8 shows the frequency distribution histogram of the input parameters and the shear capacity of the UHPC beam.

**Table 5:** Statistical analysis of the database.

Input parameter	Sign	Data type	Maximum value	Minimum value	Mean value	SD
Shear span ratio	**λ**	Input	4.50	0.80	2.05	0.75
Cross-section width	b (mm)	Input	200.00	50.00	129.00	44.38
Cross-section height	h (mm)	Input	650.00	76.00	291.45	142.54
Compressive strength of UHPC	*f*_c_ (MPa)	Input	154.50	80.80	125.18	12.28
Stirrup ratio	*ρ*_SV_ (%)	Input	4.50	0.00	0.34	0.74
Volume fraction of steel fiber	*V*_f_ (%)	Input	3.20	0.00	1.72	0.87
Shear strength of UHPC beam	*V*_ex_ (kN)	Output	1164.00	71.00	473.33	217.55

## Methodology

### Artificial neural network (ANN)

**Figure 9:**
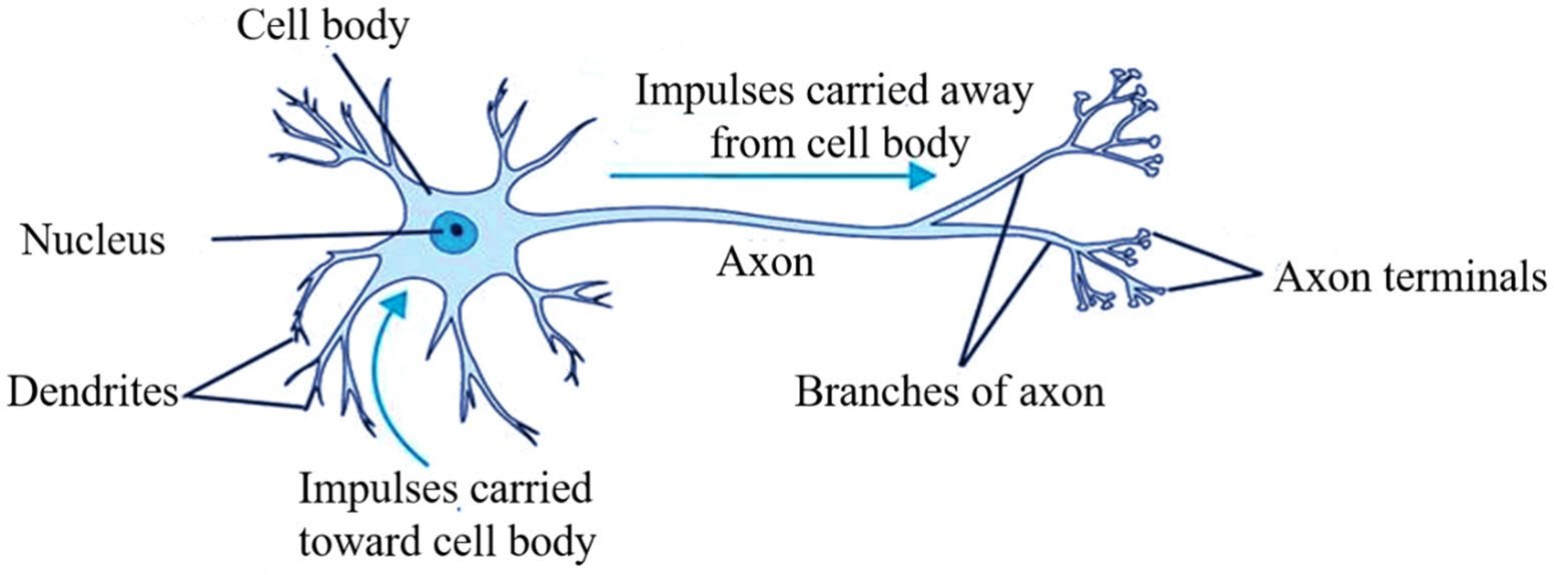
Schematic diagram of biological neurons.

**Figure 10:**
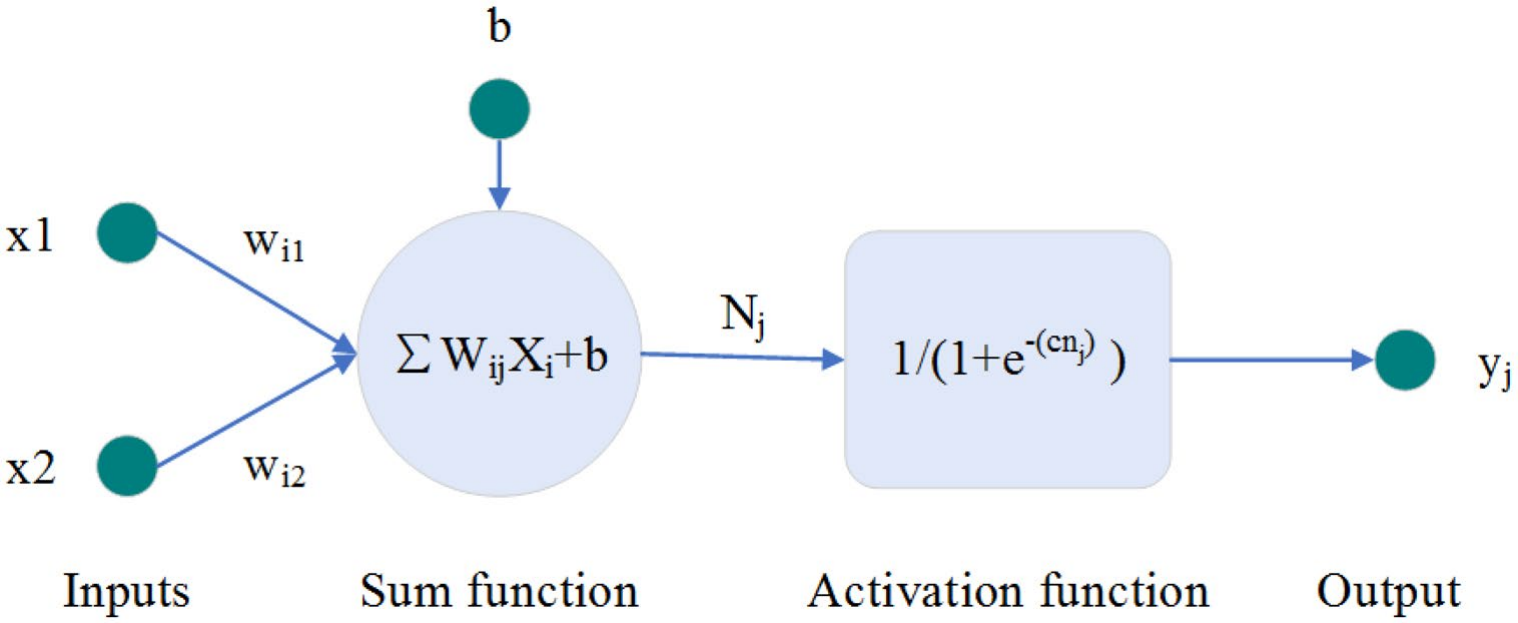
Perceptron structure.

The human brain is composed of many interconnected neurons working together^57^, and the ANN simulates the structure of human brain neurons, as shown in Fig. 9. The perceptron is the simplest ANN structure and is composed of neurons with two inputs and one output^58^. The perceptron performs a weighted summation of the data and then adds a threshold as the input of the activation function. The structure of the perceptron is shown in Fig. 10. X represents the input, w_ij_ represents the weight, and the weighted sum is calculated by formula (1). Activation functions usually have step, linear, ramp, hyperbolic tangent, sigmoid functions, and ReLU functions^59^. In this study, we chose log-sigmoid, tan-sigmoid, and Purelin functions as activation functions, which are defined by Eqs. (2)–(4) as follows:

1 $$ {\text{N}}_{{\text{j}}} = \sum\limits_{{{\text{i}} = 1}}^{{\text{n}}} {{\text{W}}_{{{\text{ij}}}} {\text{X}}_{{\text{i}}} + {\text{b}}} $$

2 $$ {\text{Log-sigmoid:}}\;{\text{g}}({\text{x}}) = \frac{1}{{1 + {\text{e}}^{{ - {\text{x}}}} }} $$

3 $$ {\text{Tan-sigmoid:}}\;{\text{g(x) = }}\frac{2}{{1 + {\text{e}}^{{ - {\text{x}}}} }} - 1 $$

4 $$ {\text{Purelin:}}\;{\text{g}}({\text{x}}) = {\text{x}} $$

**Figure 11:**
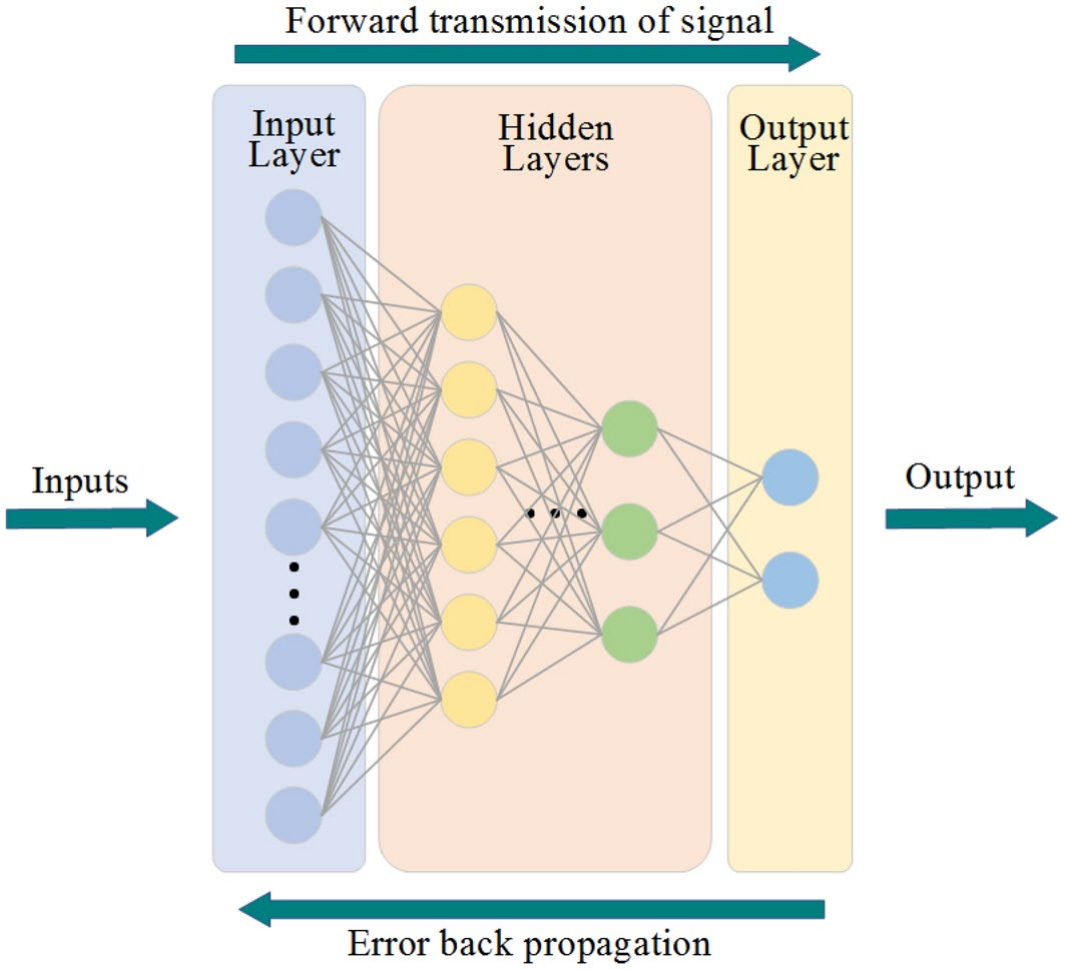
Multilayer perceptron structure.

The most common learning algorithm of ANNs is back propagation (BP). The implementation process of the BP algorithm is divided into two steps: (I) The input layer receives the input information and transmits it to the output layer and then compares the output value with the target value to determine the error; (II) the error is propagated back, and the “generalized incremental rule” is used to adjust the weight and threshold of the network to reduce the total error through the error gradient descent method. The above training process is performed in cycles until the output error reaches the set target value. The multilayer perceptron is the most common ANN structure. Its structure is shown in Fig. 11. The left side is the input layer, which obtains the input data. One or many hidden layers are in the middle, including most of the core processing units of the ANN. On the right is the output layer of the ANN, which outputs the results we want^60^. The BP algorithm can reduce the training time of the ANN, so this study chooses the BP neural network (BPNN).

### Genetic algorithm (GA)

The GA is the most commonly used algorithm to solve optimization problems^61^. The emergence of the GA is inspired by natural selection and biological evolution mechanisms. A GA takes all individuals in a group as objects and uses randomization technology to efficiently search an encoded parameter space. Among them, selection, crossover, and mutation constitute the genetic operation of the genetic algorithm. The core content of the genetic algorithm includes five elements: parameter coding, initial population setting, fitness function design, genetic operation design, and control parameter setting. Individuals in a population represent chromosomes and are sorted by the selected fitness function. Retain high-fitness individuals, so the new offspring group performance is higher than that of the father. The above process is repeated until the conditions meet and the fitness function reaches the maximum.

### Genetic algorithm-backpropagation neural network (GA-BPNN)

**Figure 12:**
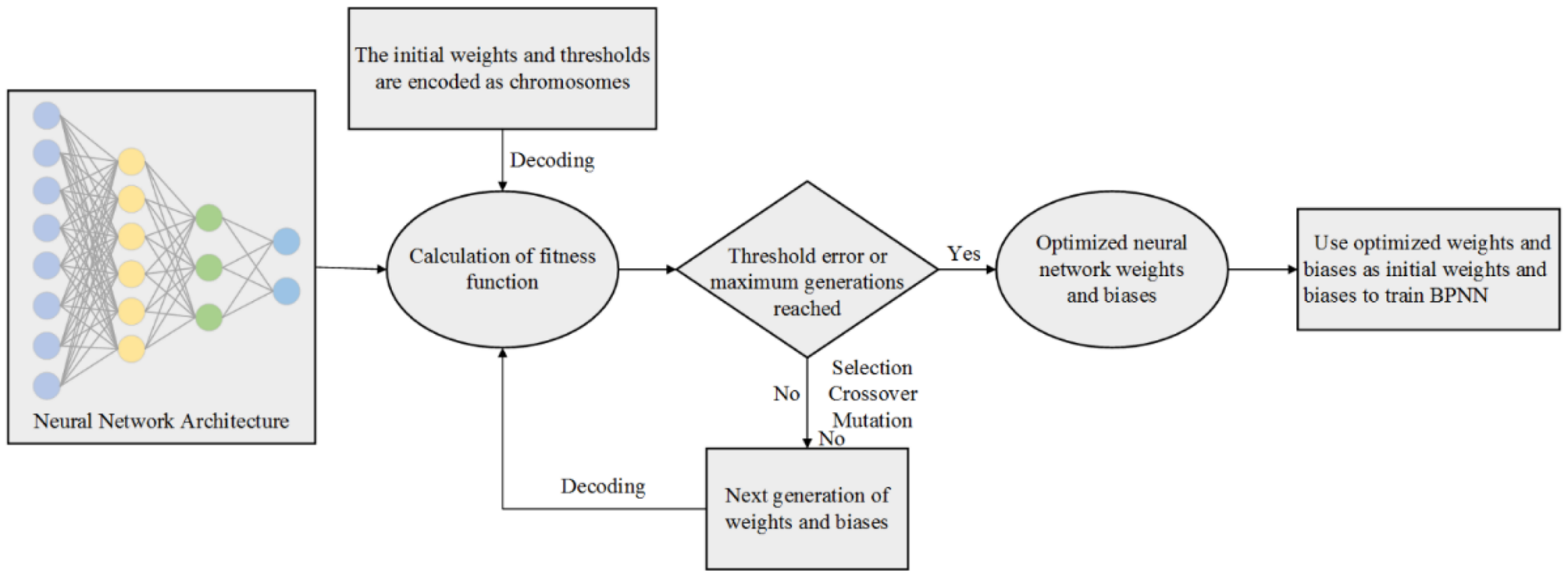
GA-BPNN flow chart.

The global search ability of a GA helps to optimize the initial weights and thresholds of a BPNN, thereby enhancing the robustness of the BPNN. The process of combining a GA with a BPNN is divided into two steps. The first step is to find the optimal initial weight and threshold of the BPNN by the global searching ability of the GA. In the second step, the optimal weights and thresholds obtained by the GA-BPNN are used to train the network in combination with the BP algorithm. The fitness function of the genetic algorithm considers the mean square error (MSE), which is defined as follows: Fig. 12 shows the process of the GA-BPNN.

5 $$ {\text{f}}({\text{w}}_{{\text{i}}} ,{\text{b}}_{{\text{i}}} ) = \frac{1}{{\text{n}}}\sum\nolimits_{{{\text{h}} = 1}}^{{\text{n}}} {\left[ {\sum\nolimits_{{{\text{m}} = 1}}^{{\text{k}}} {\left\{ {{\text{t}}_{{{\text{hm}}}} - {\text{p}}_{{{\text{hm}}}} \left( {{\text{w}}_{{\text{i}}} ,{\text{b}}_{{\text{i}}} } \right)} \right\}^{2} } } \right]} $$

where t_hm_ represents the target value, p_hm_ is the predicted value based on the weight w_i_ and the threshold bi, n is the number of training samples, and k is the number of samples for the predicted value.

### Model performance verification

Three statistical parameters were selected to evaluate the accuracy of the model, including the Pearson correlation coefficient R^2^, mean square error MSE and root mean square error RMSE. R^2^ measures the fitting degree between the predicted and experimental values, and MSE and RMSE represent the deviation between the predicted and experimental results. The three statistical parameter formulas are as follows^62^ (6)–(8):

6 $$ {\text{R}}^{2} = 1 - \left( {\frac{{\sum\nolimits_{{{\text{i}} = 1}}^{{\text{n}}} {({\text{x}}_{{\text{i}}} - {\text{y}}_{{\text{i}}} )^{2} } }}{{\left( {{\text{x}}_{{\text{i}}} - {\text{x}}} \right)^{2} }}} \right) $$

7 $$ {\text{MSE = }}\frac{1}{{\text{n}}}\sum\nolimits_{{{\text{i}} = 1}}^{{\text{n}}} {\left( {{\text{x}}_{{\text{i}}} - {\text{y}}_{{\text{i}}} } \right)^{2} } $$

8 $$ {\text{RMSE = }}\sqrt {\frac{1}{{\text{n}}}\sum\nolimits_{{\text{i = 1}}}^{{\text{n}}} {\left( {{\text{x}}_{{\text{i}}} - {\text{y}}_{{\text{i}}} } \right)^{2} } } $$

In the formula, n is the number of data sets, x_i_ is the predicted value of the model, and y_i_ is the experimental value.

Among them, RMSE is the most commonly used statistical error parameter and directly compares the predicted value with the experimental value. An RMSE of 0 indicates that the predicted value is identical to the experimental value^63,64^. R^2^ is based on the linear relationship between the predicted and experimental values, and R^2^ = 1 represents the best correlation between the predicted and experimental values^65^.

To evaluate the reliability of the ANN model, the engineering index a20-index is introduced:

9 $$ {\text{a}}20 - index{ = }\frac{m20}{M} $$

where M is the number of dataset samples and m20 is the number of samples with the ratio of the experimental value/predicted value falling between 0.80 and 1.20.

### K-fold validation

**Figure 13:**
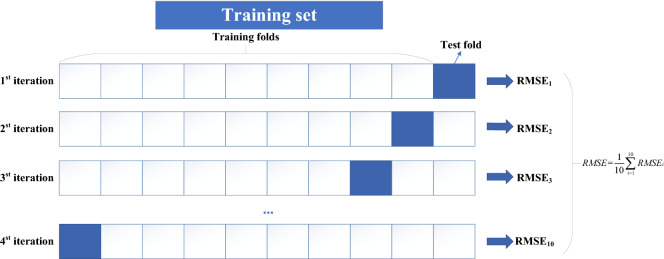
K-fold cross-validation.

To determine the best neural network parameters, the training data are divided into a training set and a verification set. Due to data contingency, dividing different validation sets and data sets may obtain different results. The best way to solve this problem is K-fold cross-validation. K-fold cross-validation is generally used for model tuning to find the hyperparameters that make the model’s generalization performance optimal. After determining the parameters, the model was retrained on all training sets, and the independent test set was used to evaluate the model performance. The data were divided into K partitions. The model was trained on K−1 partitions, and the same model was cross-trained. Finally, the average performance of the model was obtained. In this study, K = 10, and K-fold cross-validation is shown in Fig. 13.

### Data standardization

Data standardization processing is the key step of neural network technology and can greatly improve the learning efficiency of ANNs. It can eliminate the difference in dimensions between different data^66,67^ and control the data range within [0,1]. The standardized processing formula is shown in Eq. (10).

10 $$ {\text{X}}_{{\text{n}}} = \frac{{{\text{X}} - {\text{X}}_{\min } }}{{{\text{X}}_{\max } - {\text{X}}_{\min } }} $$

In the formula, X_n_ represents the standardized value, Xmin represents the minimum value of each variable, and Xmax represents the maximum value of each variable.

### BPNN structure design

Determining the best BPNN structure is a key link in establishing a BPNN model. Currently, no specific criteria are available for determining the structure of a BPNN, and many scholars use trial and error to determine the structure^68–70^. In this study, a four-layer BPNN model was created using MATLAB R2016a. To determine the optimal number of hidden layer neurons, 1200 BPNNs were developed. We ranked 1200 BPNNs with RMSE values to determine the best BPNN structure.

## Results

### Best BPNN structure

**Figure 14:**
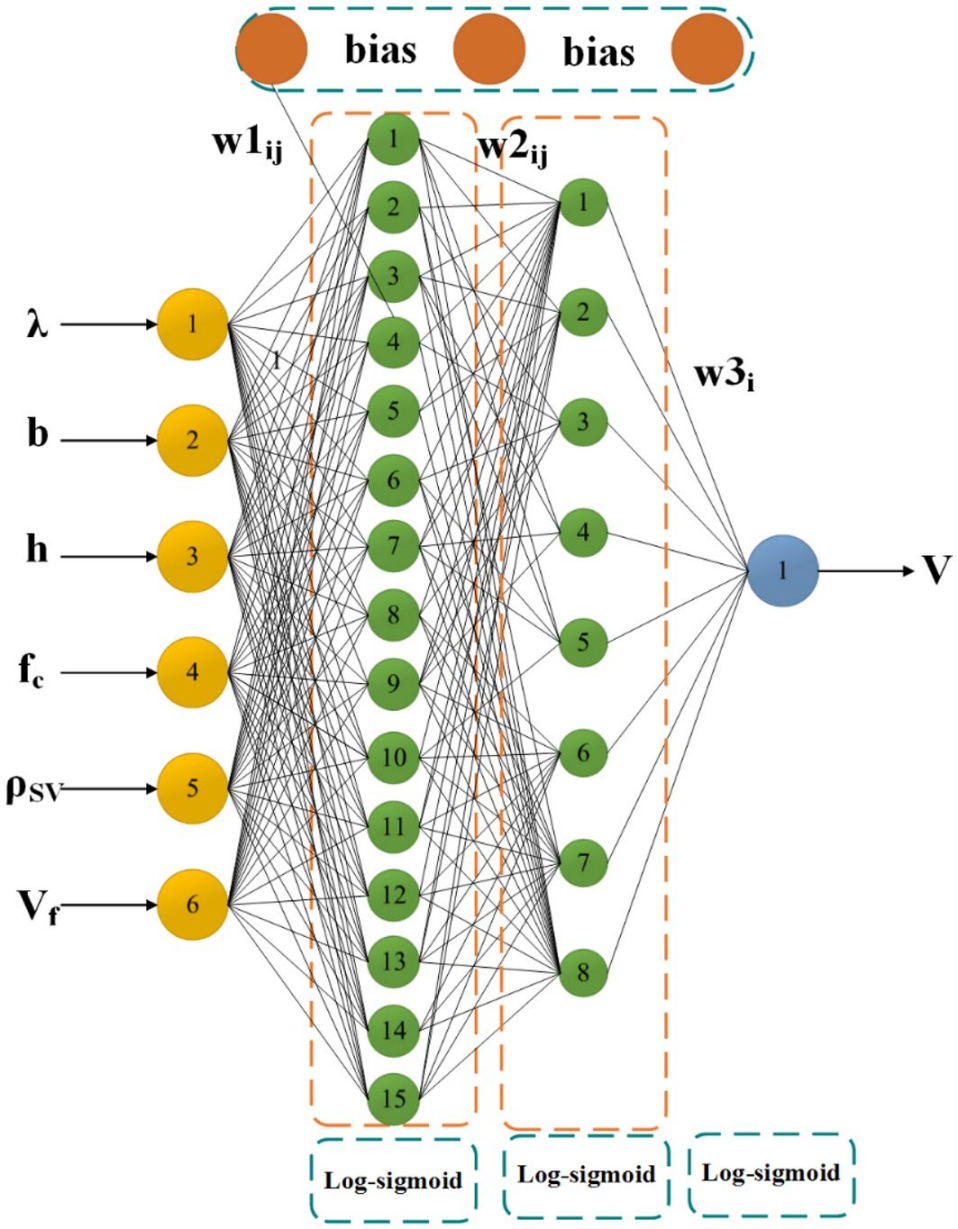
Schematic diagram of the optimal BPNN structure (6-15-8-1).

**Figure 15:**
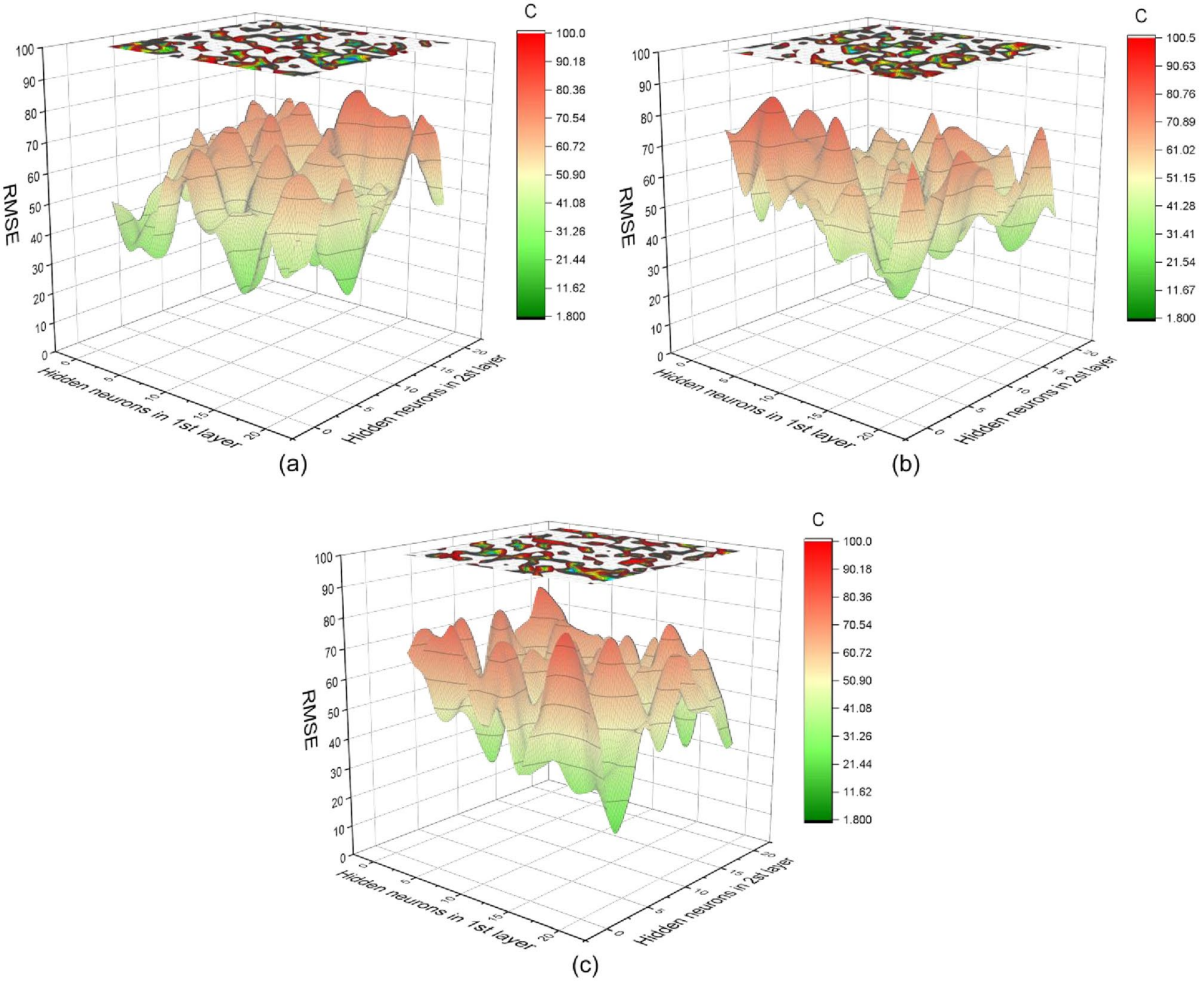
RMSE values for different BPNN models {(**a**) Log-sigmoid, (**b**) Tan-sigmoid, (**c**) Purelin}.

The optimal BPNN structure was determined by trial and error, and 1200 BPNN models were trained by setting the number of neurons in different hiding layers and different activation functions. The structure with the lowest RMSE value was the optimal BPNN structure. The parameter settings of the BPNN are shown in Table 6. Each model was trained with 72 data points (70.59%) and tested with 30 data points (29.41%). Each model was ranked according to the RMSE value. Table 7 lists the best 10 BPNNs, and the optimal BPNN structure is 6-15-8-1. The optimal BPNN structure is shown in Fig. 14. Figure 15 shows the RMSE values of the 1200 BPNN models.

**Table 6:** BPNN parameter settings.

Parameter	Setting
Training algorithm	Gradient descent method
Number of hidden layers	2
Number of hidden layer neurons	1–20
Epochs	500
Activation function	Log-sigmoid, Tan-sigmoid, Purelin
Model performance	R^2^, RMSE, a20-index

**Table 7:** Ranking of BPNN models based on RMSE (top 10).

Ranking	Structures	Activation function	RMSE	R^2^	a20-Index
1	6-15-8-1	Log-sigmoid	8.08	0.96164	0.9036
2	6-17-17-1	Log-sigmoid	8.16	0.90927	0.8632
3	6-18-16-1	Log-sigmoid	8.70	0.92044	0.8732
4	6-14-15-1	Log-sigmoid	8.76	0.85431	0.8625
5	6-18-6-1	Log-sigmoid	9.15	0.90205	0.8865
6	6-9-13-1	Log-sigmoid	9.28	0.94201	0.8568
7	6-3-5-1	Log-sigmoid	9.38	0.85001	0.8326
8	6-7-18-1	Tan-sigmoid	9.41	0.87488	0.9025
9	6-20-20-1	Tan-sigmoid	9.45	0.91159	0.9125
10	6-18-13-1	Log-sigmoid	9.48	0.89275	0.8936

### GA-BPNN model

**Figure 16:**
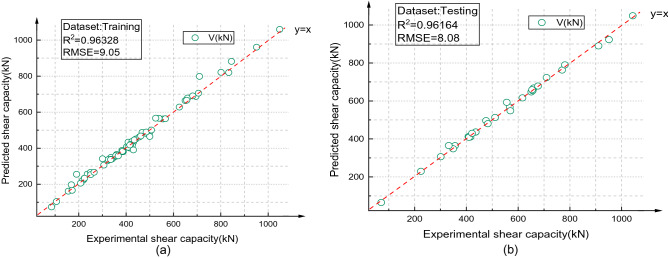
BPNN (6-15-8-1) prediction results {(**a**) Training set, (**b**) Testing set}.

**Figure 17:**
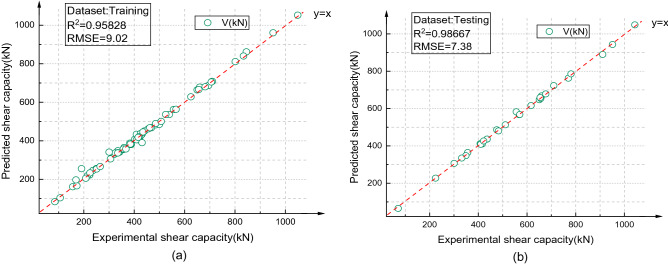
GA-BPNN prediction results {(**a**) Training set, (**b**) Testing set}.

The best BPNN structure (6-15-8-1) was determined, and the output result of BPNN (6-15-8-1) is shown in Fig. 16. The correlation coefficient (R^2^) between the predicted value and the test value of the test set was 0.96164, and the RMSE value was 8.08. The output results of the GA-BPNN are shown in Fig. 17. The correlation coefficient (R^2^) between the predicted values and the test values of the test set was 0.98667, and RMSE = 7.38. The results showed that the prediction performance of the GA-BPNN was better than that of the BPNN.

### Comparison of GA-BPNN with existing design specifications

**Figure 18:**
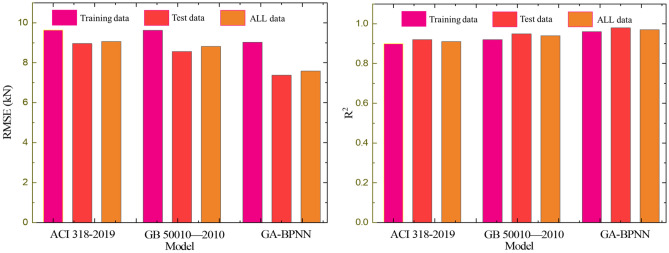
Statistical results of model performance.

The calculation results of the existing specifications were compared with the established GA-BPNN model. The existing specifications that are more applied were selected: the Specification for the Design of Concrete Structures (GB50010-2010)^71^ and the American Code for the Design of Concrete Structures (ACI 318-2019)^72^. The predicted performance was quantified, and the chosen quantifiers were the root mean square error (RMSE) and correlation coefficient (R2). The comparison results are shown in Fig. 18, and it can be seen that GA-BPAA shows better results.

### Comparison of GA-BPNN with other methods

**Figure 19:**
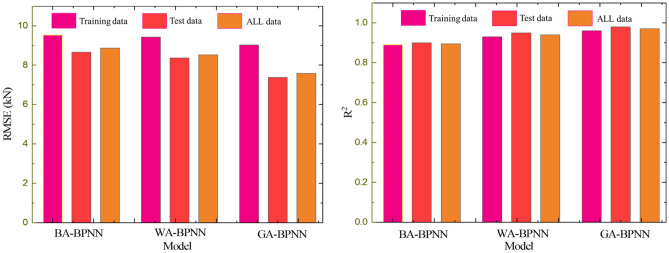
Comparison of GA-BPNN, BA-BPNN, WA-BPNN.

The bat algorithm (BA) and whale algorithm (WA) are introduced to compare with the optimization results of the GA. BA is a stochastic search algorithm that simulates the use of a type of sonar by bats in nature to detect prey and avoid obstacles, i.e., it simulates the most basic detection and localization ability of bats using ultrasound for obstacles or prey and relates it to the optimization of target functions^73^. The whale optimization algorithm (WOA) is an algorithm based on the behavior of whales that round up prey^74^. The comparison results are shown in Fig. 19.

## Discussion

### Advantages of the GA-BPNN

**Figure 20:**
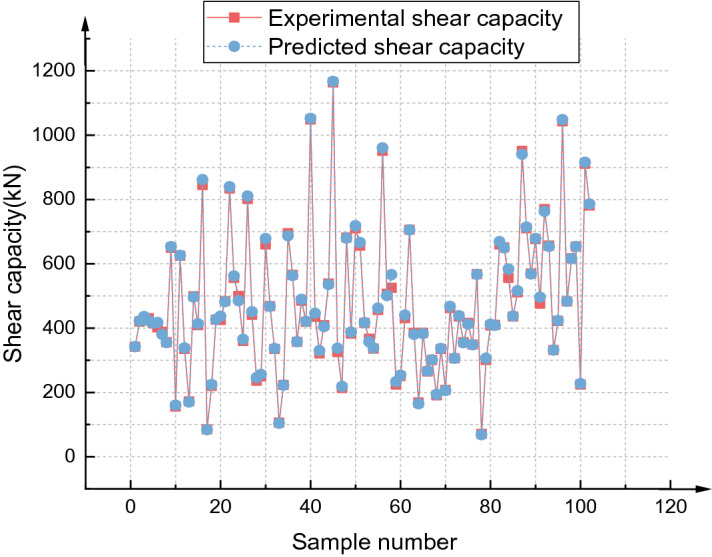
GA-BPNN fitting effect.

**Figure 21:**
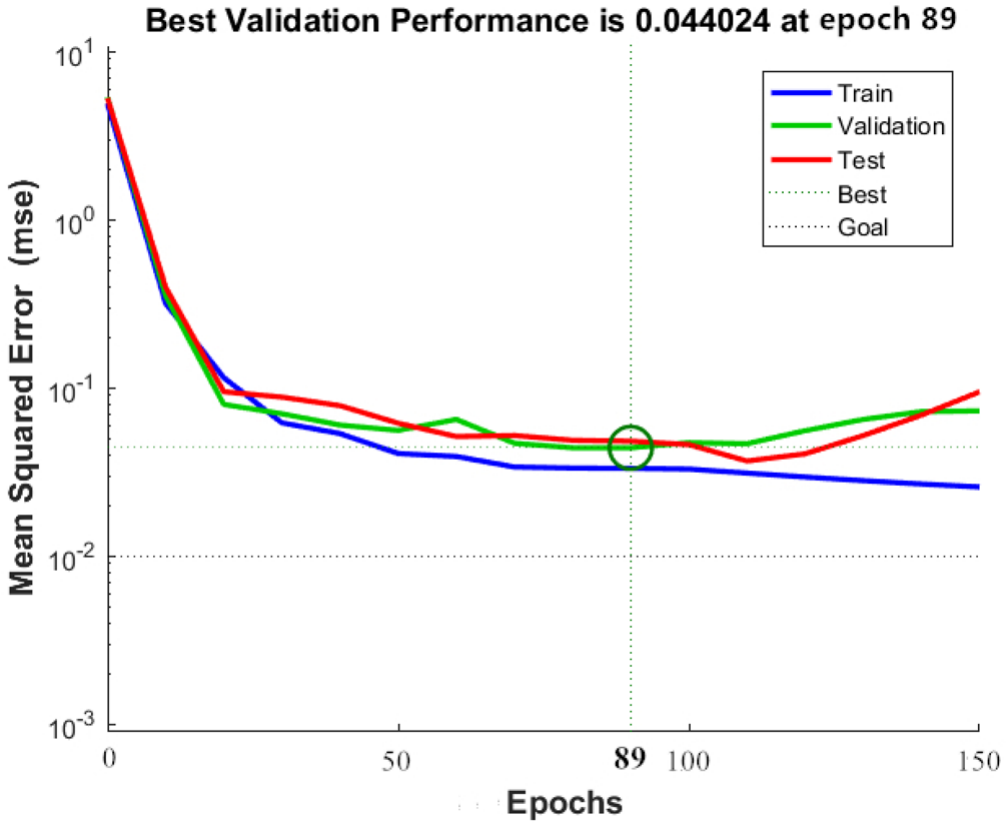
Error change of the GA-BPNN iterative process.

The initial weight and threshold of the BPNN are optimized by the GA, which solves the problem that the BP algorithm easily falls into local optima and increases the convergence speed of the BPNN. In terms of statistical parameters, after GA optimization, R^2^ increased from 0.96164 to 0.98667, and the RMSE decreased from 8.08 to 7.38. The fitting effect between the predicted value of the GA-BPNN and the experimental value is shown in Fig. 20. Figure 21 shows the process of error reduction in the iteration process of the GA-BPNN. The figure describes the change in network error with the number of iterations when the network is trained with the training set. In the figure, the blue line represents the error of the training set, the red line represents the error of the test set, and the green line represents the error of the validation set. When the error of the validation set no longer decreases, the training stops. The training speed of the GA-BPNN is much faster than that of the BPNN, and the training stops in the 89th generation.

### Sensitivity analysis

Determining the relationship between each input variable and output variable in an ANN is very important^75^ and can enhance the understanding of the relationship between input parameters and output parameters in the model. Sensitivity analysis is usually used to determine the influence of input variables on output in a mathematical model. In this study, the method proposed by Milne^76^ was used for the sensitivity analysis of six input variables, and the formula is as follows (11):

11 $$ {\text{IIF = }}\frac{{\sum\nolimits_{{{\text{j}} = 1}}^{{{\text{n}}_{{{\text{hidden}}}} }} {\frac{{{\text{w}}_{{{\text{ji}}}} }}{{\sum\nolimits_{{{\text{l}} = 1}}^{{{\text{n}}_{{{\text{inputs}}}} }} {\left| {{\text{w}}_{{{\text{j}}1}} } \right|} }}} {\text{w}}_{{{\text{oj}}}} }}{{\sum\nolimits_{{{\text{k}} = 1}}^{{{\text{n}}_{{{\text{inputs}}}} }} {\left( {\sum\nolimits_{{{\text{j}} = 1}}^{{{\text{n}}_{{{\text{inputs}}}} }} {\left| {\frac{{{\text{w}}_{{{\text{jk}}}} }}{{\sum\nolimits_{{{\text{l}} = 1}}^{{{\text{n}}_{{{\text{inputs}}}} }} {\left| {{\text{w}}_{{{\text{j1}}}} } \right|} }}{\text{w}}_{{{\text{oj}}}} } \right|} } \right)} }} $$

where *IIF*represents the importance of the input parameters, W represents the connection weights between neurons, W_ij_ represents the connection weights between the input layer and the hidden layer, W_oj_ represents the connection weights between the output layer and the hidden layer, *l*, *I*, *k* represents the input layer neurons, hidden layer neurons, and output layer neurons, respectively, and *n*_*inputs*_ represents the number of input parameters and hidden neurons.

**Figure 22:**
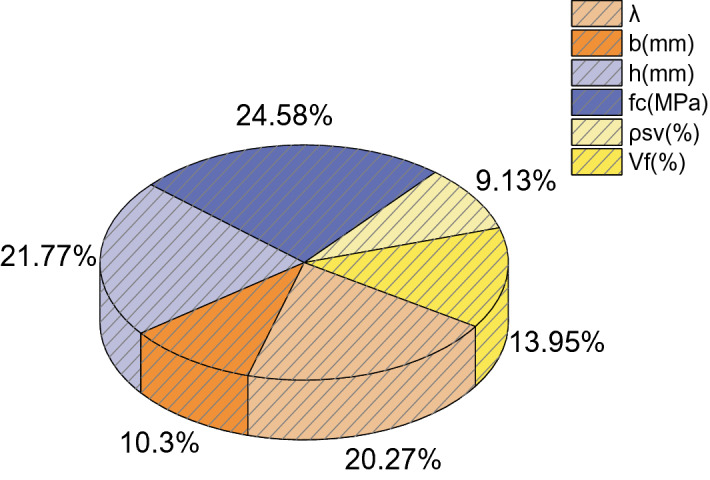
Sensitivity analysis results.

Figure 22 shows the results of the sensitivity analysis. The compressive strength of UHPC has the greatest influence on the shear bearing capacity of UHPC beams, and its contribution factor is 24.58%. The stirrup ratio has the smallest influence, and its contribution factor is 9.13%. The order of contribution factors from large to small is UHPC compressive strength *f*_c_ > beam cross-section height (h) > shear span ratio (λ) > steel fiber volume fraction (*V*_f_) > beam cross-section width (b) > stoop ratio (*ρ*_SV_). The sensitivity analysis results show that the six selected parameters have a greater impact on the shear capacity of the beam, and all selected parameters are representative. The results of the sensitivity analysis can provide a reference for designers.

## Conclusions

In this study, a new type of high-strength UHPC beam was proposed, and six UHPC beams were tested under static loading to study the shear performance of UHPC beams. To quantify the shear capacity of UHPC beams, a machine learning method is used to establish a prediction model of the shear capacity of UHPC beams. The following conclusions are obtained:

- Through the static load test of six UHPC beams, it is found that the shear strength of UHPC beams with steel fiber is improved to a great extent. Steel fibers in UHPC can act in concert with shear reinforcement to suppress shear cracks.
- Through literature research, it is found that most of the existing models for predicting the shear capacity of UHPC beams adopt empirical formulas for regression. This method has strong limitations and is applicable only to the established experimental conditions and not to most possible situations, so it is difficult to apply under complex engineering conditions.
- Based on the experimental data of 102 UHPC beams in this study and previous articles, the shear span ratio (λ), beam section width (b), beam section height (h), stirrup ratio (*ρ*_SV_), UHPC compressive strength (*f*_c_) and steel fiber volume fraction (*V*_f_) were used as input parameters. The BPNN model with two hidden layers was constructed to predict the shear capacity of UHPC beams. The correlation coefficient (R^2^) between the predicted value and the experimental value was 0.96164, and the RMSE was 8.08.
- Using a GA to optimize the initial weights and thresholds of a BPNN, the results show that the prediction performance of the GA-BPNN is better than that of the BPNN. Its prediction accuracy is higher, R^2^ increased from 0.96164 to 0.98667, and the RMSE decreased from 8.08 to 7.38.
- A sensitivity analysis of the selected input parameters is performed to determine the importance of the input variables. The order of contribution factors is UHPC compressive strength (*f*_c_) > beam cross-sectional height (h) > shear span ratio (λ) > steel fiber volume fraction (*V*_f_) > beam cross-sectional width (b) > stirrup ratio (*ρ*_SV_).

## Limitations and future directions

The data in the database established in this study were obtained under different experimental conditions. Because of the differences in experimental equipment and human operation behaviors, the relevance of research among different scholars decreases, which increases the prediction error of the model and slows down the iteration time. The model may not be accurate for ranges beyond the dataset; our following study will extend our data with finite element tools to apply the model to more comprehensive applications.

## Supplementary Information

Supplementary Information.

## Data Availability

The data are available in this article.
