# Supplementary material for: Prediction of the shear capacity of ultrahigh-performance concrete beams using neural network and genetic algorithm
Source: Sci Rep. 2023 Feb 7;13:2145. doi: 10.1038/s41598-023-29342-0 (PMC9905517; doi:10.1038/s41598-023-29342-0)
Supplement: Supplementary file 1 — Supplementary Information. [file 41598_2023_29342_MOESM1_ESM.docx]

**Appendix Table 1.** Test database.

| **Data Source (Literature)** | **Specimen Number** | **λ** | **b (mm)** | **h (mm)** | ***f*_c_ (MPa)** | ***ρ*_SV_ (%)** | ***V*_f_ (%)** | ***V*_ex_ (KN)** |
| --- | --- | --- | --- | --- | --- | --- | --- | --- |
| This study | UHPC-1 | 1.00 | 200.00 | 300.00 | 110.00 | 3.20 | 0.00 | 660.00 |
|  | UHPC-2 | 1.50 | 200.00 | 300.00 | 110.00 | 3.20 | 1.00 | 710.00 |
|  | UHPC-3 | 1.50 | 200.00 | 300.00 | 125.00 | 3.20 | 2.00 | 625.00 |
|  | UHPC-4 | 2.00 | 200.00 | 300.00 | 125.00 | 0.00 | 3.00 | 570.00 |
|  | UHPC-5 | 2.00 | 200.00 | 300.00 | 150.00 | 4.50 | 0.00 | 525.00 |
|  | UHPC-6 | 2.00 | 200.00 | 300.00 | 110.00 | 2.50 | 1.00 | 650.00 |
| Voo et al. [48] | X-B1 | 3.20 | 50.00 | 650.00 | 125.00 | 0.00 | 1.00 | 660.00 |
|  | X-B2 | 3.20 | 50.00 | 650.00 | 126.00 | 0.00 | 1.00 | 710.00 |
|  | X-B3 | 3.20 | 50.00 | 650.00 | 135.00 | 0.00 | 1.00 | 483.00 |
|  | X-B4 | 2.50 | 50.00 | 650.00 | 122.00 | 0.00 | 1.00 | 911.00 |
|  | X-B5 | 3.50 | 50.00 | 650.00 | 140.00 | 0.00 | 1.00 | 845.00 |
|  | X-B6 | 4.50 | 50.00 | 650.00 | 140.00 | 0.00 | 1.00 | 781.00 |
|  | X-B7 | 2.50 | 50.00 | 650.00 | 122.00 | 0.00 | 1.50 | 1043.00 |
|  | X-B8 | 1.80 | 50.00 | 650.00 | 122.00 | 0.00 | 1.00 | 1164.00 |
| Haibin et al. [49] | L1 | 3.13 | 120.00 | 350.00 | 120.00 | 0.26 | 1.50 | 408.00 |
|  | L2 | 2.49 | 120.00 | 350.00 | 125.00 | 0.26 | 1.50 | 499.00 |
|  | L3 | 1.50 | 120.00 | 350.00 | 125.00 | 0.26 | 1.50 | 1049.00 |
|  | L4 | 2.09 | 120.00 | 350.00 | 123.00 | 0.65 | 1.50 | 677.00 |
|  | L5 | 2.62 | 120.00 | 350.00 | 128.00 | 0.44 | 1.50 | 568.00 |
|  | L6 | 2.09 | 120.00 | 350.00 | 124.00 | 0.44 | 1.50 | 654.00 |
|  | L7 | 1.73 | 120.00 | 350.00 | 124.00 | 0.26 | 1.50 | 706.00 |
|  | L8 | 2.08 | 120.00 | 350.00 | 100.00 | 0.44 | 1.50 | 431.00 |
|  | L9 | 2.08 | 120.00 | 350.00 | 100.00 | 0.65 | 1.50 | 511.00 |
| Lingzhi et al. [50] | L1 | 1.51 | 150.00 | 250.00 | 117.20 | 0.00 | 2.00 | 656.50 |
|  | L2 | 1.75 | 150.00 | 250.00 | 117.20 | 0.00 | 2.00 | 385.00 |
|  | L3 | 2.20 | 150.00 | 250.00 | 117.20 | 0.00 | 2.00 | 356.25 |
|  | L4 | 3.02 | 150.00 | 250.00 | 117.20 | 0.00 | 2.00 | 335.00 |
|  | L5 | 2.20 | 150.00 | 250.00 | 117.20 | 0.17 | 2.00 | 419.25 |
|  | L6 | 2.20 | 150.00 | 250.00 | 117.20 | 0.25 | 2.00 | 430.85 |
|  | L7 | 2.20 | 150.00 | 250.00 | 117.20 | 0.58 | 2.00 | 426.25 |
|  | L8 | 2.20 | 150.00 | 250.00 | 117.20 | 0.00 | 2.00 | 300.50 |
|  | L9 | 2.20 | 150.00 | 250.00 | 117.20 | 0.00 | 2.00 | 420.00 |
|  | L10 | 2.20 | 150.00 | 250.00 | 117.20 | 0.00 | 1.00 | 249.75 |
|  | L11 | 2.20 | 150.00 | 250.00 | 117.20 | 0.00 | 3.00 | 415.50 |
| Qiang et al. [51] | L-0 | 2.26 | 150.00 | 250.00 | 80.80 | 0.00 | 2.00 | 356.00 |
|  | LA-1 | 1.51 | 150.00 | 250.00 | 96.27 | 0.00 | 2.00 | 651.00 |
|  | LA-2 | 1.75 | 150.00 | 250.00 | 101.23 | 0.00 | 2.00 | 385.00 |
|  | LA-3 | 3.02 | 150.00 | 250.00 | 117.20 | 0.00 | 2.00 | 335.00 |
|  | LB-1 | 2.26 | 200.00 | 350.00 | 117.20 | 0.17 | 2.00 | 422.00 |
|  | LB-2 | 2.26 | 200.00 | 350.00 | 117.20 | 0.25 | 2.00 | 431.00 |
|  | LB-3 | 2.26 | 200.00 | 350.00 | 125.30 | 0.45 | 2.00 | 435.00 |
|  | LB-4 | 2.26 | 200.00 | 350.00 | 119.40 | 0.75 | 2.00 | 485.00 |
|  | LC-3 | 2.26 | 200.00 | 350.00 | 132.10 | 0.00 | 3.00 | 416.00 |
|  | LD-1 | 2.26 | 200.00 | 350.00 | 125.30 | 0.00 | 2.00 | 301.00 |
|  | LD-2 | 2.26 | 200.00 | 350.00 | 125.30 | 0.00 | 2.00 | 425.00 |
|  | LE-1 | 2.26 | 200.00 | 350.00 | 125.30 | 0.00 | 2.00 | 325.00 |
| Zongcai et al. [52] | B1-80-1.5 | 1.30 | 120.00 | 150.00 | 116.80 | 0.25 | 1.00 | 321.10 |
|  | B1-80-2.5 | 2.17 | 120.00 | 150.00 | 115.80 | 0.25 | 1.00 | 224.50 |
|  | B1-80-3.5 | 3.03 | 120.00 | 150.00 | 112.70 | 0.25 | 1.00 | 190.70 |
|  | B1-130-1.5 | 1.30 | 120.00 | 150.00 | 115.10 | 0.38 | 1.00 | 306.00 |
|  | B1-130-2.5 | 2.17 | 120.00 | 150.00 | 117.60 | 0.38 | 1.00 | 236.20 |
|  | B1-130-3.5 | 3.03 | 120.00 | 150.00 | 118.90 | 0.56 | 1.00 | 168.70 |
|  | B1-200-1.5 | 1.30 | 120.00 | 150.00 | 114.30 | 0.56 | 1.00 | 250.70 |
|  | B1-200-2.5 | 2.17 | 120.00 | 150.00 | 116.50 | 0.25 | 1.00 | 220.50 |
|  | B1-200-3.5 | 3.03 | 120.00 | 150.00 | 114.80 | 0.25 | 1.00 | 156.00 |
|  | B1-130-2.5 | 2.17 | 120.00 | 150.00 | 118.50 | 0.25 | 1.00 | 213.90 |
|  | 1B1-130-2.5 | 2.17 | 120.00 | 150.00 | 117.10 | 0.38 | 1.00 | 224.40 |
|  | 2B1-80-2.5 | 3.03 | 120.00 | 150.00 | 125.70 | 0.38 | 1.00 | 223.10 |
| Aziz et al. [53] | B181U | 1.00 | 120.00 | 180.00 | 134.50 | 0.00 | 1.00 | 951.30 |
|  | B1815U | 1.50 | 120.00 | 180.00 | 134.50 | 0.00 | 1.00 | 833.60 |
|  | B182U | 2.00 | 120.00 | 180.00 | 134.50 | 0.00 | 1.00 | 207.90 |
|  | B241U | 1.00 | 120.00 | 240.00 | 134.50 | 0.00 | 1.00 | 801.20 |
|  | B2415U | 1.50 | 120.00 | 240.00 | 134.50 | 0.00 | 1.00 | 681.60 |
|  | B242U | 2.00 | 120.00 | 240.00 | 134.50 | 0.00 | 1.00 | 264.80 |
|  | B301U | 1.00 | 120.00 | 300.00 | 134.50 | 0.00 | 1.00 | 951.30 |
|  | B3015U | 1.50 | 120.00 | 300.00 | 134.50 | 0.00 | 1.00 | 616.90 |
|  | B302U | 2.00 | 120.00 | 300.00 | 134.50 | 0.00 | 1.00 | 656.10 |
| Lim et al. [54] | SB1 | 3.00 | 150.00 | 290.00 | 115.00 | 0.00 | 1.50 | 172.00 |
|  | SB2 | 3.00 | 150.00 | 290.00 | 115.00 | 0.60 | 1.50 | 408.90 |
|  | SB3 | 3.00 | 150.00 | 290.00 | 115.00 | 0.90 | 1.50 | 441.00 |
|  | SB4 | 3.00 | 150.00 | 290.00 | 115.00 | 1.40 | 1.50 | 436.10 |
| Pu et al. [55] | L-4-100-a | 4.00 | 60.00 | 430.00 | 144.50 | 0.47 | 1.00 | 360.00 |
|  | L-3-100-a | 3.00 | 60.00 | 430.00 | 132.70 | 0.47 | 1.00 | 410.00 |
|  | L-2-100-a | 2.00 | 60.00 | 430.00 | 145.10 | 0.47 | 1.00 | 556.00 |
|  | L-1-100-a | 1.00 | 60.00 | 430.00 | 152.50 | 0.47 | 1.00 | 770.00 |
|  | L-1-100-a-8 | 1.00 | 60.00 | 430.00 | 127.10 | 0.84 | 1.00 | 695.00 |
|  | L-2-0-a | 2.00 | 60.00 | 430.00 | 148.90 | 0.00 | 1.00 | 484.00 |
|  | L-2-200-a | 2.00 | 60.00 | 430.00 | 134.30 | 0.24 | 1.00 | 500.00 |
|  | L-2-150-a | 2.00 | 60.00 | 430.00 | 154.50 | 0.31 | 1.00 | 507.00 |
|  | L-2-150-b | 1.99 | 60.00 | 426.79 | 143.50 | 0.31 | 1.00 | 539.00 |
|  | L-2-150-c | 2.01 | 60.00 | 418.75 | 151.20 | 0.31 | 1.00 | 566.00 |
|  | 1-1-100-a | 1.00 | 60.00 | 430.00 | 133.90 | 0.47 | 1.00 | 556.00 |
|  | 1-3-100-a | 3.00 | 60.00 | 430.00 | 128.40 | 0.47 | 1.00 | 456.00 |
| Pourbaba et al. [56] | B1a | 1.20 | 152.00 | 152.00 | 137.00 | 0.00 | 3.20 | 416.00 |
|  | B1b | 1.20 | 152.00 | 152.00 | 137.00 | 0.00 | 3.20 | 476.00 |
|  | B2a | 1.20 | 152.00 | 152.00 | 137.00 | 0.00 | 3.20 | 468.00 |
|  | B2b | 1.20 | 152.00 | 152.00 | 137.00 | 0.00 | 3.20 | 436.00 |
|  | B3a | 1.20 | 152.00 | 152.00 | 137.00 | 0.00 | 3.20 | 462.00 |
|  | B3b | 1.20 | 152.00 | 152.00 | 137.00 | 0.00 | 3.20 | 357.00 |
|  | B4a | 1.20 | 152.00 | 152.00 | 137.00 | 0.00 | 3.20 | 367.00 |
|  | B4b | 1.20 | 152.00 | 152.00 | 137.00 | 0.00 | 3.20 | 388.00 |
|  | B5a | 1.20 | 152.00 | 152.00 | 137.00 | 0.00 | 3.20 | 402.00 |
|  | B5b | 1.20 | 152.00 | 152.00 | 137.00 | 0.00 | 3.20 | 383.00 |
|  | B21 | 1.20 | 152.00 | 152.00 | 125.00 | 0.00 | 3.20 | 349.00 |
|  | B22 | 1.20 | 152.00 | 152.00 | 125.00 | 0.00 | 3.20 | 332.00 |
|  | B23 | 1.20 | 152.00 | 152.00 | 125.00 | 0.00 | 3.20 | 335.00 |
|  | B24 | 1.20 | 152.00 | 152.00 | 125.00 | 0.00 | 3.20 | 336.00 |
|  | B29 | 0.90 | 102.00 | 203.00 | 125.00 | 0.00 | 3.20 | 409.00 |
|  | B30 | 0.80 | 102.00 | 203.00 | 125.00 | 0.00 | 3.20 | 342.00 |
|  | B35 | 2.80 | 152.00 | 76.00 | 125.00 | 0.00 | 3.20 | 106.00 |
|  | B36 | 2.80 | 152.00 | 76.00 | 125.00 | 0.00 | 3.20 | 85.00 |
|  | B37 | 2.70 | 152.00 | 76.00 | 125.00 | 0.00 | 3.20 | 71.00 |
